# Mindfulness Training in UK Secondary Schools: a Multiple Case Study Approach to Identification of Cornerstones of Implementation

**DOI:** 10.1007/s12671-018-0982-4

**Published:** 2018-06-22

**Authors:** Stephanie Wilde, Anna Sonley, Catherine Crane, Tamsin Ford, Anam Raja, James Robson, Laura Taylor, Willem Kuyken

**Affiliations:** 1 Department of Psychiatry, Warneford Hospital, University of Oxford, Oxford OX3 7JX, UK; 2 Medical School, University of Exeter, Exeter, UK; 3 Department of Education, University of Oxford, Oxford, UK

**Keywords:** Implementation, Mindfulness, Secondary schools, Qualitative research, Focus groups, Semi-structured interviews

## Abstract

This paper examined the facilitators and barriers to implementation of mindfulness training (MT) across seven secondary/high schools using a qualitative case study design. Schools varied in level of implementation. Within schools, head teachers, members of school senior leadership teams, and staff members involved in the implementation of MT were interviewed individually. In addition, focus groups were conducted with other members of school staff to capture a broad range of views and perspectives. Across the case studies, several key themes emerged, which suggested four corner stones to successful implementation of MT in schools. These were: *people*, specifically the need for committed individuals to champion the approach within their schools, with the support of members of the senior leadership teams; *resources*, both time and financial resources required for training and delivery of MT; *journey*, reflecting the fact that implementation takes time, and may be a non-linear process with stops and starts; and finally *perceptions*, highlighting the importance of members of the school community sharing an understanding what MT is and why it is being introduced in each school context. Similarities and differences between the current findings and those of research on implementation of other forms of school mental health promotion programs, and implementation of MT in healthcare settings, are discussed.

## Introduction

Increasing concern about the mental health of adolescents has been met by the development of programs for young people that promote mental well-being and develop life skills (Sawyer et al. 2012). Schools are often seen as the primary setting where such efforts should be focused, because of their broad reach and central role in the lives of children and families (Greenberg 2010). Recent systematic reviews and governmental reports suggest that universal approaches, offered to a whole school community, have the greatest potential to promote the mental health of young people (Vostanis et al. 2013; Weare and Nind 2011). However, for such universal interventions to succeed, they need to be implemented effectively with appropriate attention to facilitators and barriers to implementation (Durlak and DuPre 2008; Merry 2012).

Mindfulness is commonly defined as the “awareness that emerges through paying attention on purpose, in the present moment, and nonjudgmentally to the unfolding of experience moment to moment” (Kabat Zinn 2003, p. 144). Mindfulness programs for schools, and for young people more broadly, aim to cultivate this capacity through a range of activities often including experiential mindfulness practices, psychoeducation, and cognitive-behavioral exercises. For the purposes of this study, we use the term Mindfulness Training (MT) to refer to the provision of explicit teaching directed towards the cultivation of mindfulness.

A number of school-based MT programs exist (see Meiklejohn et al. 2012). Evidence to date, derived primarily from studies that are relatively small in scale and variable in methodological quality, suggests that MT programs for youth are associated with small but significant enhancements on a range of social-emotional (e.g., negative emotion, distress, pro-social behavior), cognitive (e.g., meta-cognition and cognitive flexibility), and behavioral (e.g., academic achievement and school functioning) outcomes (Klingbeil et al. 2017). Work specifically reviewing MT in schools has likewise identified significant small to-moderate effects of MT on students’ cognitive performance, stress, and resilience (Zenner et al. 2014).

Despite these promising findings, systematic reviews point to the need for larger, well-controlled studies with longer periods of follow-up (e.g., Klingbeil et al. 2017; Maynard et al. 2017). In addition, greater attention to intervention fidelity and implementation of MT is required. Reflecting this need, a recent review of the current research literature on school-based mindfulness and yoga interventions has demonstrated that most do not report fidelity of program implementation beyond issues of participant dosage (e.g., Feagans Gould et al. 2016).

Although, as highlighted above, there is relatively little research that addresses the implementation of MT programs in schools, this limitation is not unique to this setting. Indeed a recent review of mindfulness science more broadly has mapped existing research across translational stages, from basic science, through feasibility studies, to pilot trials, largerscale effectiveness trials, and on to implementation (Dimidjian and Segal 2015). The review suggests that across the broad field of mindfulness science, most research is at the feasibility/pilot stage, and for the field to realize its potential public health impact, there is a need to ensure the full spectrum of translational research is conducted. The review also highlights that the relative absence of research into the implementation of mindfulness-based interventions (the “implementation cliff”) has the potential to lead to mindfulness interventions “stalling out” (Dimidjian and Segal 2015, p.608).

Ensuring that new scientific knowledge is effectively translated into activities that have an impact in the real world involves challenges and opportunities (Rycroft-Malone and Bucknall 2010). Some implementation challenges will be shared across many intervention domains and contexts. Others may be specific to mindfulness interventions as a whole (Dimidjian and Segal 2015), or to mental health promotion in schools (Durlak and DuPre 2008). Finally, there may be some implementation barriers and facilitators that are unique to mindfulness programs delivered in educational settings. Exploring these common facilitators and barriers alongside any unique features is therefore likely to support development of the most comprehensive understanding of the implementation of MT in school settings.

The large body of work on the implementation of other forms of mental health promotion programs in schools suggests that high-quality implementation is an essential condition of effective Social and Emotional Learning (SEL) programs, which are designed to improve the mental health, well-being, and/or social and emotional competencies of young people (e.g., Durlak 2015). Further, it is suggested that program outcomes cannot be interpreted fully without also investigating the process of implementation. For example, it has been demonstrated across 213 studies (*N* = 270,034 children and adolescents) that attention to implementation produces a stepwise enhancement in the effectiveness of schoolbased mental health promotion programs (Durlak et al. 2011). Existing studies and reviews have identified as many as 23 factors that influence implementation, which can be summarized as including community-level influences, characteristics of the staff, and features of the school (e.g., Cooper et al. 2015; Domitrovich et al. 2008; Durlak 2015; Durlak 2016; Durlak and DuPre 2008; Fixsen et al. 2005; Greenhalgh et al. 2005). More specifically, the support and engagement of the school leadership, the training of key school staff, successful collaboration among multiple stakeholders, the ability to adapt the program in question both to the school context and any broader policy context, and the availability of requisite administrative and financial resources are all regarded as critical. Finally, in order to ensure that these factors come together to produce a good outcome, appropriate monitoring of the quality of implementation is required (Durlak 2015). Although this research addresses implementation of SEL more broadly, many of these factors are also likely to be highly relevant to the specific case of implementation of mindfulness programs in schools.

Work in healthcare settings, the context in which many existing mindfulness-based programs originated, has also elucidated categories of facilitators/barriers to effective implementation (Eccles et al. 2009; Grimshaw et al. 2012; Nilsen 2015). Indeed, a recent study has examined the facilitators and barriers to the implementation of mindfulness-based cognitive therapy (MBCT) in the UK National Health Service (Rycroft-Malone et al. 2014). This study used the PARiHS framework (Promoting Action on Research Implementation in Health Services; Kitson et al. 2008) as a heuristic device to inform the study’s design, data collection, and analysis. This framework suggests that successful implementation can be conceptualized as the product of the nature and type of evidence (both scientific and more informal) available to support the intervention being implemented, the qualities of the context in which the intervention in question is being implemented, and the process of facilitation (e.g., the factors that support and expediate the implementation process). Using a two-phase qualitative, exploratory, and explanatory case study approach, Rycroft-Malone et al. suggested that (1) despite widespread interest, access to MBCT remains very limited and variable in the UK National Health Service, illustrating the challenge of implementation and (2) sustainable implementation is a process and a journey, often over many years. The study findings indicated that in the UK health service implementation of MBCT was often facilitated “bottom up” by “champions,” who were often very skilled, committed, and resourceful individuals who created networks, organized training, adapted MBCT to the needs of the local context and at key “pivot points” in the implementation journey saw, and seized opportunities. Sustainability over many years was often supported by identifying a niche, adapting and enhancing MBCT to fit the niche, and then building capacity, first through grassroots bottom up support and in time through top-down management support. Capacity building requires careful consideration of models of training and supervision and proactive succession planning. Although many of these factors are shared with other forms of implementation (e.g., Pearson et al. 2015), the importance of both MT champions, who were instrumental in the implementation journey, and the associated grassroots bottom up support these champions nurtured, were identified as features distinctive to MBCT implementation. It is unknown whether similar factors are also important in the implementation of mindfulness-based programs in other settings, including schools.

In this study, we were interested in furthering the understanding of implementation of MT across a range of UK schools. Within each school, we explored the perspectives of different stakeholders in the implementation process: speaking to head teachers, members of school senior leadership teams, staff members involved in the implementation of MT, and other staff members with little or no direct experience, including those with skeptical attitudes. Our aim was to develop an understanding of the journey towards implementation within each case school and to explore the common themes that emerged across these cases, using inductive thematic analysis to identify themes emerging in the data, with the inclusion of codes derived deductively from the PARiHS framework, described above. In summary, the objectives of the study were to, first, identify key facilitating factors and potential barriers to implementation of mindfulness in schools, and second, consider the extent to which any of the identified facilitators and barriers are (a) unique to school-based MT interventions or (b) shared with other relevant interventions and contexts.

## Method

### Participants

We sought to identify a pool of secondary/high schools implementing MT. Identification occurred through discussion with mindfulness training centers, Internet searches, and word of mouth. Through this search process, relatively few schools could be identified that were offering formalized provision of MT within their curriculum. SW contacted all schools that were identified to gauge eligibility, and schools that had not yet begun any MT work with pupils, or whose provision had completely ceased, were excluded at this point. Of the remaining schools, seven schools participated (around half of those identified and approached) and these were selected to represent a mixture of different *school types*: state-funded, independent, selective, non-selective; a range of *geographical locations*: both urban and rural; a range of *socioeconomic contexts* as determined by proportion of pupils eligible for free school meals relative to national averages; a range of school *quality ratings*, based on Office for Standards in Education (OFSTED), England, ratings where available; and a range of *stages on the implementation journey*. This spread of school types and contexts was intended to increase the likelihood that we would obtain the perspectives of staff in schools that were facing different internal and external pressures, and that might differ in their rationale for wishing to implement mindfulness. Characteristics of participating schools are shown in Table 1.

#### Participants Identification

Within each case school, data were collected through semi-structured interviews with key stakeholders (the head teacher, other members of the school senior leadership team, SLT, such as deputy or associate head teachers, and the mindfulness lead) and focus groups with other members of staff, to ensure that a plurality of perspectives was represented. Initial contact with schools was usually through the mindfulness teachers (mindfulness leads), and in all cases, these leads then assisted with setting up interviews with the head teachers and other SLT members. Focus groups were also set up with the support of the mindfulness leads, who passed on study information to members of staff to ensure that as far as possible each group included people representing a range of degrees of engagement with mindfulness training, including sampling teachers who were broadly supportive of mindfulness and those who were more critical.

#### Sociodemographic Characteristics and Exposure to Mindfulness

All participants were between 21 and 65 years of age. Across the sample as a whole, 36 female and 42 male staff members participated. These staff held a range of roles and there were varying degrees of use of and exposure to mindfulness both within and across schools. Table 2 outlines the composition of participants within each school, their roles and their exposure to mindfulness.

### Procedure

A multiple case study design was adopted (Stake 1995; Yin 2014). This design was intended to provide an in-depth understanding of the implementation journey of the seven secondary schools. In each case, the data collection included individual semi-structured interviews with key stakeholders in the implementation process (the school mindfulness lead who was the teacher taking responsibility for mindfulness delivery in the school and members of the SLT) and focus groups with members of the wider teacher population to provide multistakeholder insights into the implementation of MT in each case school and a space for debate, discussion, and disagreement about MT in schools. Neither mindfulness leads nor head teachers were present in the focus groups, to allow participants to speak freely. Interviews and focus groups took place on site in each school and were undertaken and facilitated by members of the research team (SW, AS, and LT). In all cases, the mindfulness lead was interviewed, excluding River, where a particular mindfulness lead could not be identified and Leafy, where the mindfulness lead supported the research and provided information informally but was not formally interviewed. One focus group was held at each school apart from Leafy where three focus groups were held due to a larger number of participants.

The study protocol was reviewed by the University of Oxford Ethics Committee (MS-IDREC-C1–2015-063). The research was conducted in accordance with the ethical framework produced by the British Educational Research Association (BERA 2011). Information sheets about the research were distributed to participants in advance of fieldwork visits and consent forms were signed either before or at the beginning of data collection sessions. Case study schools and individual participants have been anonymized and where direct quotations have been presented, identifying information has been removed, to maintain the anonymity of participants and schools.

### Measures

Separate topic guides (included in Appendices 1 and 2) were used in interviews and focus groups and examined: why and how MT was introduced; its perceived benefits/costs; approaches to implementation at different stages of preparation and sustainability; barriers and facilitators of implementation, both those experienced and those anticipated; and perspectives on what resources might support schools in implementing MT. The interviews enabled in-depth exploration of these issues, while the focus groups, with a broader array of participants, allowed multiple views to be explored simultaneously, further stimulating discussion and enabling a plurality of perspectives to be sampled through exploration of group discussion. All interviews and focus groups were audio-recorded and transcribed. Background information was gathered from publicly available documents and other sources such as school websites, and field notes were systematically taken by the team as they researched the schools during the case selection process, undertook site visits, and conducted interviews and focus groups. This additional data source provided valuable contextual information for each case and was therefore included in the analysis to aid interpretation of the data provided directly by interviewees and focus group participants.

### Data Analyses

Thematic analysis was used to identify and explore key patterns relevant to the research aims (Braun and Clarke 2006). The four data sources, interview transcripts, focus group transcripts, background materials, and field notes, were analyzed together. Field notes and background materials were used to provide a broader context within which the interview and focus group transcripts could be interpreted. Data from each case study were analyzed separately to ensure an indepth understanding of the cases was established before cross-case analysis took place. This approach enabled the researchers to examine the perspectives of stakeholders in the implementation journey across a range of different school contexts in the UK with cross-case analysis allowing for the identification of common facilitators and barriers across these different schools.

Thematic analysis was structured by six key phases: becoming familiar with the data, generating initial codes by annotating transcripts, searching for themes, reviewing themes, defining and naming themes, and producing an analytical report (Braun and Clarke 2006). This process was primarily conducted by SW, who has extensive training and experience. However, codes and themes were validated through cross-coding by two additional researchers, AS and WK (Saldaña 2012; Sandelowski and Barroso 2007). This analysis was undertaken concurrently but interdependently with the three team members meeting at fortnightly intervals to discuss the process.

Initial codes were developed inductively, emerging from the data on a case by case basis. These were then cross referenced with codes derived deductively using the PARiHS explanatory framework as a heuristic device, reflecting the significance of *evidence* for the benefits of mindfulness, the importance of *context*, and the process of *facilitation*. Codes were grouped to identify subthemes, inter-theme relationships were examined, and convergences and divergences explored. This led to the development of four meta-themes, referred to here as the four key cornerstones: the *people* involved in mindfulness, the *resources* made available for mindfulness, the *implementation journey*, and *perceptions* of mindfulness. These emerged through cross-case analysis and the combination of both inductive and deductive codes and higher level patterns (Miles et al. 2014). Member checking (Lincoln and Guba 1985) then took place through a focus group to which key stakeholders from across the seven schools were invited, alongside a number of other individuals involved in the implementation of mindfulness in schools in the UK. At this focus group, participants were invited to comment on the findings and themes that emerged after the initial analysis at the aforementioned focus group meeting with the research team.

Every effort was made to ensure the study was undertaken with appropriate rigor. Tracy’s (2010) eight criteria for “excellent qualitative research” (selection of a worthy topic, rich rigor, sincerity, credibility, resonance, significant contribution, ethical conduct, and meaningful coherence) and Lincoln and Guba’s (1985) criteria for rigor in qualitative research (credibility, transferability, dependability, confirmability) were used to shape the design and analysis of the study. Particular emphasis was placed on ensuring the reliability and credibility of the findings through multi-stakeholder research participation, member checking the initial analysis and findings, and through independent coding by multiple researchers.

## Results

### Main Findings

The schools in the study differed from one another in a number of ways: how efforts to implement mindfulness had progressed, the stage of implementation reached, and the way in which mindfulness was being used in the schools. All schools had introduced and delivered the Mindfulness in Schools Project ‘.b’ program (https://mindfulnessinschools.org/what-is-b/b-curriculum/) or derivatives thereof, in their curricula. This is a highly structured program intended primarily for delivery by specifically trained teachers, to whole school classes, with teachers required to complete an 8-week personal mindfulness course, have a period of 6 months personal mindfulness practice, and then attend a 4-day syllabus training prior to delivery to pupils. At the time of the fieldwork, the way the program was being implemented varied from school to school. Two of the schools were delivering the MT program with a high degree of fidelity, whereas others had made significant adaptations. For example, mindfulness was not always delivered in curriculum time, but in more than one school was provided through drop-in sessions or sessions for target groups of pupils, with particular needs. One of the schools had modified an available mindfulness program and the teachers were using it without having had formal training. Despite these differences, analysis of metathemes allowed for the development of a broader understanding of those factors that facilitate or are barriers to the implementation of MT in school settings.

The four meta-themes identified by the research, *people*, *journey*, *resources*, and *perceptions*, crossed all case studies and contained both differences and similarities between schools at the subtheme level. For example, within the *perceptions* meta-theme, key subthemes included “involving teachers and communicating about mindfulness”, “using shared language”, “emphasizing what mindfulness is not,” and “recognizing benefits of mindfulness for staff, students and parents.” In some schools with established MT, there was evidence of the use of a collective language to refer to mindfulness, which was shared by staff and pupils. In contrast in other schools with less established MT, this shared language and understanding was not observed. Likewise, for the *resources* meta-theme, key subthemes included “finding curriculum space,” “allowing time for mindfulness to embed,” and “funding staff, staff training and resources.” In many schools, negotiating and sustaining curriculum time for MT was a challenge. However, the challenges imposed by the limited availability of material resources and funding for training and staff were more apparent in schools that were facing budgetary constraints. All schools could be conceptualized as on a *journey* towards implementation of MT, in which *people* played a key role both in facilitating or hindering implementation. However, the nature of this journey was individual to each school, and highly dependent on the issues captured by the other meta-themes.

Below, we describe the four meta-themes that emerged from the cross-case analysis of all seven schools, and which appeared to reflect cornerstones of successful implementation. We then discuss how these meta-themes were expressed differently across participants and schools.

### People

Within each school, key people were identified as instrumental to implementation, either through their skillful facilitation and specialist training in mindfulness teaching (typically mindfulness leads) or their position of influence within the school (typically head teachers and members of the SLT). In each school, the mindfulness lead, who was often a dedicated, committed, and enthusiastic individual (or more than one individual) was key, and implementation had faltered in the only school in the study that did not have a mindfulness lead. Additionally, having a supportive or at least not obstructive head teacher and senior leadership team underpinned effective implementation. “Your starting point is you have to have a champion. I think it depends on having somebody there who is constantly or regularly advocating its strength and is prepared to champion it” (Leafy, SLT). This was echoed in other settings: “I think the big thing is having a good person to deliver it” (Lake, Focus Group). Similarly, the importance of having support from senior leadership to enable implementation was regarded as essential:

I think from having your SLT on board you get training, you get time, you get people, you build your network because you’re allowed to go on days and meet people or whatever. But you’re only going to get SLT on board if you’ve got your evidence and your data and then only once you’ve got SLT and all that stuff going on, the attitude and the ethos. (Garden, Focus Group)

Even if SLT members were not actively supportive, a lack of active obstruction was important: “So SLT support, if not active, at least, you know, you have to have that because if there is a block you haven’t got a hope, so I suppose that’s a milestone” (Leafy, Focus Group).

One major challenge identified through our fieldwork was the fact that turnover of staff and changes in leadership within a school could result in a rapid loss of mindfulness expertise and capacity from one year to the next. For example at Garden, a number of key staff members with interest and expertise in MT, including a previous head teacher, had left the school, while at River, the member of staff who had been formally trained to deliver the MT program and had initially introduced MT to the school had left, resulting in two untrained staff members taking forward less formalized delivery of MT with substantial inclusions and adaptations. Implementation relied heavily, at least in its initial stages, on the energy, enthusiasm, and vision of mindfulness “champions” within the schools, and the departure of these champions threatened ongoing provision.

Talking about the problems arising from the departure of trained staff, one of the participants said:

One of the challenges is that if the leading members of staff, and there are not that many at the moment, decide to go elsewhere, and if we lost key figures in the school that are teaching at the moment, could we lose it? (Fields, Focus Group)

Respondents also expressed concern about the qualifications held by teachers of MT and raised issues related to safeguarding and the quality of prior training: “So it would worry me if I thought people were going on a one day inset course and then teaching mindfulness” (Fields, SLT). Another said:

So my reservations would just be about people teaching it, that they are qualified and indeed that the support structures are in place that if pupils had a negative reaction they would know how to, you know, how to help. (Leafy, SLT)

Although staff turnover is likely to affect implementation of many types of school-based programs, the relatively high-cost and time-intensive training required to deliver most school-based mindfulness programs, combined with the relative scarcity of trained teachers, means that staff turnover creates greater challenges for sustainable implementation than might be the case for other programs such as literacy interventions that do not require extensive additional teacher skill acquisition.

### Resources

A second key theme that emerged from the analysis was the importance of appropriate resource allocation to support implementation of MT in schools. Allowing sufficient space on the curriculum, and sufficient time for MT to embed within the school, was a key and was a perceived as linked to the need for a specific commitment to MT within the school, which echoes the findings related to the importance of *people*, described above. A participant in one school that had been implementing MT for a number of years, said, “another powerful lesson for us, which we always articulate to our colleagues, is if you’re going to do this you’ve got to resource it properly, and understand there is a commitment required there” (Fields, SLT).

The allocation of sufficient time and financial resources for training staff in the program, and then supporting their role in an ongoing way, posed significant challenges to effective and sustained implementation in a number of schools. A context of the intensifying accountability, and the demands on schools to raise standards in key curriculum areas and respond rapidly to policy changes, militated against the implementation of MT. This was largely due to the pressures placed on schools in terms of budget, staff time, and curriculum flexibility: “We’ve got 1,600 children to put through an eight-week timetable, and I’m not sure that we have got the capacity to do that for every child” (Meadow, SLT). Another said:

We have five inset days a year, there are huge demands on those for everybody, for increasing literacy, for numeracy, for supporting students with learning difficulties. There are new GCSEs, new A-Levels being introduced simultaneously, the demand on teachers’ time is phenomenal. (Meadow, SLT)

Other respondents raised the impact of limited financial resources and staff time. One said “but it’s the cost of getting a teacher certificated that is a barrier, without a doubt. Particularly in times of budget constraint” (Park, SLT), with another adding “but it’s the amount of time as well as the expense, as well as the cost of the course itself” (Park, SLT). Indeed, although theoretically MT might be regarded as particularly beneficial for students and staff in poorly performing schools, due to its potential impact on staff and student wellbeing and self-regulation, participants in this study felt that implementation would be extremely difficult in such circumstances and potentially unlikely to succeed.

### Journey

Participants reflected on the process of implementation within the school, from preparation for the initial introduction of MT to the time of the research. Schools described different types of implementation *journey* and where implementation had progressed, participants described involving teachers in the process, sharing experiences of mindfulness in schools, communicating with colleagues and also cooperating with other schools. Several mentioned the distinction between MT as a discrete timetabled subject and MT as an element present within the broader ethos and culture of the school. In some schools, implementation had gradually resulted in MT becoming more embedded within the school over time, whereas others described how initial enthusiasm and interest had diminished over time, weakening or almost eliminating provision of MT within the school.

So it’s really embedded and there’s a common vocabulary between pupils and staff because so many members of staff have done it. It’s something that the pupils know they do and it’s something that they can hear their older peers talking about and it’s something that has a real currency in the school. So I think it really is sustainable in its current model. (Fields, Mindfulness Lead)

Originally when I started it was very much becoming embedded, it was in most years, most teachers were talking about it. Now it’s sort of, as with most things, I think it’s deflated a little and that’s partly because we aren’t publicizing it. But it’s that kind of you do need to be mindful but also push it, it’s almost to sell it, the concept. (Garden, Mindfulness Lead)

In some schools, diminishing enthusiasm resulted from departure of a key person or key people who had been actively involved in promoting MT within the school, which highlights the relationship between the two cornerstones of *people* and *journey.* In other schools, challenges concerning how to increase the status of mindfulness in school, particularly concerning staff involvement were raised. For example:

Yes, I think the main one is: How do you get your staff on board? And, do you allow it to grow through champions, and people who pilot it, and want to be enthusiasts, and do you allow it to have a partial existence in the school, and is it okay to do that? (River, SLT)

It’s a case of, you don’t necessarily get whole-staff buyin, because of the fads. They’ve got faculty buy-in, and it’s working really well, there might be something else that comes in, and will mindfulness go, I don’t know, is my honest answer. I think, with the current model, it’s plateaued, is my critical sense. (River, SLT)

The shift from mindfulness as a niche activity at a school to a curricular element and embedded feature of the school culture requires ongoing momentum and commitment. In some schools, respondents identified that there had been changes to the way in which mindfulness was perceived within the school and these *perceptions* were linked to the journey towards MT taken by the schools.

There were no challenges in the sense that people were quite open to doing it. As a bolt-on, that’s easy, to find a meeting slot is easy. So that initial momentum wasn’t difficult, I don’t think. The problem comes, for me, about how you genuinely make it part of the curriculum, for both students and for staff, when there is increasing pressure. (Meadow, SLT)

### Perceptions

The final meta-theme related to *perceptions* of mindfulness within schools. Participants in one school described how, as implementation progressed, a shared language around MT had developed, that emphasized the collective nature of mindfulness, which could be used among staff and pupils and was valued within the school. Positive perceptions of mindfulness included the view that it was something that could benefit staff and student well-being and promote the “whole person” enhancing the mental health of young people as well as improving attainment. Implementation was also supported when there was a clear perception of what mindfulness was *not*, with participants talking about mindfulness not being Buddhism “by stealth,” therapy, or requiring young people to confront pain and distress in an unsafe way.

Although positive perceptions of MT clearly facilitated implementation, participants also raised questions about the differing and sometimes conflicting rationales for implementing mindfulness within schools, as well as some skepticism about the potential benefits of mindfulness, and the role of schools in addressing mental health and well-being more generally. Referring to the plurality of messages about mindfulness one of the participants said: “People are either ill-informed or they’ve got their own version of it. They don’t like being told that doing something will help them” (Lake, Mindfulness Lead). Others raised the issue of evidence: “I mean, you want something that’s tangible, but I just can’t work out what evidence exists, really, in terms of, what statistical research evidence exists that you can just go, right, I can pick that up and go.” (River, Focus Group).

The 64,000-dollar question, of course, is, is this impacting on hard data. Is it actually impacting on the students’ progress, and [I admit] I’m skeptical that you’ll be able to produce direct evidence, because clearly, students’ performance is based on a whole host of complex interrelated factors, and being able to separate one as being a key determinant (Meadow, SLT)

Another raised the issue of adverse effects which might arise as a result of teaching mindfulness in schools: “Are you actually forcing them to almost to come up with issues that cause them stress and, you know, ruminating and all that?” (Leafy, Focus Group). While questions were also voiced about whether teaching mindfulness was a legitimate use of school time. “There’s just a risk at the moment, with it moving forward, that this group becomes a caricature. You all sit around thinking about clearing your mind? That sounds fun. Why don’t you teach them something?” (Park, SLT).

These findings mirror other research on the implementation of mindfulness in schools, that has also highlighted the potential impact of stereotypes and misperceptions on teacher buyin (e.g., Dariotis et al. 2017) and the broader suggestion of Durlak (2015, 2016) that obtaining genuine buy-in from staff is a key component of successful implementation. It is likely that where widespread misperceptions of mindfulness exist within a school community, this would pose a serious threat to such buy-in and the likelihood of successful implementation.

## Discussion

This study adopted a multiple case study design (Stake 1995; Yin 2014), with the aim of providing an in-depth understanding of barriers and facilitators to the implementation of MT across seven secondary schools at different stages of their implementation journey. The four cornerstones of implementation of MT in schools, which arose as discrete meta-themes within the analysis, were nevertheless closely linked, with significant interplay between them. Some of the meta-themes, for example those referring to the *journey* towards implementation and the role of key *people* in the implementation process, emerged more strongly in individual interviews with those in senior school leadership positions or instrumental in the introduction of mindfulness within their school. In contrast, discussions of *perceptions* of mindfulness came out more strongly in focus groups, where a plurality of perspectives were deliberately included to stimulate discussion. Issues related to *resources* were commonly raised in both individual interviews and focus groups.

The findings of our interviews and focus groups echo those of previous work on implementation of mental health promotion in schools. Many of the factors shown to be critical to successful implementation (Durlak 2002; Durlak 2015; Durlak 2016) were also evident in our work. The findings regarding *people*, *resources* and *journey* were consistent with the factors which reviews of the implementation of mental health promotion programs have argued are essential (Cooper et al. 2015; Domitrovich et al. 2008; Durlak 2015; Durlak 2016; Durlak and DuPre 2008; Fixsen et al. 2005; Greenhalgh et al. 2005); particularly having a program champion, strong leadership, engagement of the school leadership team, the training of key school staff, the ability to access the requisite administrative and financial resources, and perceived need for the program from other staff members. Interestingly, systematic evaluation of MT provision was not observed in any of the schools studied. Since monitoring and evaluation are recognized as important elements of the journey towards sustainable implementation of mental health promotion interventions (e.g., Durlak 2015), this appears to be an area in which schools might benefit from specific support. Such evaluation would ideally move beyond assessing pupil reported outcomes in the short term to following them longer term, as young people transition to further study, and the world of work and adulthood. Additionally, it would ideally include monitoring the fidelity with which programs are delivered (e.g., Feagans Gould et al. 2016) in order to identify ways to build on provision and enhance impact within each particular school context.

The study also sought to explore the extent to which facilitators and barriers to implementation of MT in schools were similar to those identified in a study of implementation of MBCT within the UK health service, which had drawn on the PARiHS framework and its key elements: *evidence*, *context*, and *facilitation*. Subthemes relevant to each of these PARiHS elements were identified and fell across the four meta-themes, or cornerstones of implementation, in the current study. The first element, *evidence*, was mentioned by a number of participants, and fell largely within the *perceptions* meta-theme, although it was also relevant to the *resources* metatheme, where evidence was seen as something that might leverage greater resources to support MT. A number of teachers referred to the current lack of research evidence for mindfulness in school settings and this appeared to pose a barrier to acceptance of mindfulness within some schools, or by some stakeholders. Evidence of a more informal, anecdotal and practice-based type, as well as evidence internal to each school, also played a role, but a number of participants commented on the potential usefulness of “hard data” on the benefits of mindfulness for young people. The perceived lack of a robust evidence base for the use of MT in schools was combined with a lack of clarity about MT’s purposes and expected outcomes, and misperceptions of MT. These findings may reflect the wide range of claims made about the effects of MT, including spurious or exaggerated claims, the array of outcomes assessed in research studies to date (e.g., cognitive, emotional, behavioral, and social outcomes) and the broad range of ways that mindfulness training is claimed to be beneficial to young people. This lack of clarity is evident in the findings of a recent systematic review of studies of school-based mindfulness and yoga interventions, which showed that less than 10% had specified core program components or proposed logic models of change, linking intervention elements to anticipated outcomes (Feagans Gould et al. 2016).

Data from the interviews and focus groups suggested that contextual factors were the key to implementation. That is to say, where the context (setting, culture and resources) was supportive of MT, implementation progressed more effectively. Within the schools, these contextual factors fell partly within the *people* meta-theme, and included the support of at least one member of the SLT, and ideally the head teacher, as well as a good “fit” with the school ethos. In addition, SLT members, at the state-funded schools in particular, indicated that mindfulness could more easily be introduced if the school was doing well in terms of its attainment and behavior management. In more challenging circumstances, such as working to improve a low school quality rating, mindfulness was perceived by the majority of senior leaders interviewed to be something that would be less likely to be developed. For schools in these more challenging circumstances, context appeared to intersect with the *resources* meta-theme to determine the likelihood that time and funding might be allocated to MT rather than to other more areas perceived to be more pressing priorities.

Hindering contextual factors, mentioned at all the schools, included the pressures on the timetable and the need to justify the use of curriculum and school time for mindfulness (reflected in the *resources* meta-theme), seeing mindfulness as a “fad” and potentially one of many short-lived educational initiatives and the challenge of establishing who mindfulness is for (reflected in the *perceptions* meta-theme), with some schools moving from targeted to more widespread provision and others moving from more widespread provision to provision focused on particular groups seen as more likely to benefit. The current climate of accountability was also influential. Indeed, while context was reflected in the cornerstones of *people*, *resources*, and *perceptions* of mindfulness, the broader legislative and sociopolitical landscape in which schools operate and which influences their decision-making about how to allocate resources was also seen as relevant.

A number of key factors, which fell across all four cornerstones, were identified that potentially facilitated and expedited the implementation of mindfulness in schools, thereby leading to more sustainable implementation of mindfulness. Mirroring the findings of the ASPIRE study (Rycroft-Malone et al. 2017), the presence of a driven individual (the mindfulness lead) or a network led by such an individual was important in most/all schools. Implementers were not only committed, but very skilled in change management, using a range of strategies and activities tailored to match the context and audience. Creating curriculum space and having an ongoing SLT commitment to mindfulness, which had the potential to survive staff changes appeared to be important in schools where MT implementation had progressed. SLT support helped secure funding for ongoing training of staff and capacity building, rather than schools relying on just one pivotal mindfulness lead. Implementation appeared to be more effective where mindfulness was offered as a regular and constant presence alongside opportunities for staff to experience mindfulness. It was also facilitated where strategies existed for responding effectively to resistance from teachers and pupils, and there was cooperation with other schools, for example by communicating about how best to implement mindfulness and sharing training costs and venues.

Again, many of the factors highlighted above are common to the broader implementation of mental health promotion interventions in schools or to the implementation of mindfulness in healthcare settings. The ASPIRE findings demonstrated that while initial implementation was often driven by one or two champions in a bottom up way, over time top-down and properly resourced facilitation was required for sustainability (Rycroft-Malone et al. 2017). Gradually, service champions had developed networks at different levels of the health service and had enabled changes in perceptions, culture, and service ethos (Rycroft-Malone et al. 2017). At two of the schools that had been offering MT for almost 10 years, such changes were also evident and were discussed by the head teachers during the interviews in very positive terms, with particular regard to the contribution of MT to the ethos of the schools. Finally, the ASPIRE study also identified “pivot points,” periods where implementation could either surge forwards or falter. These included staff arriving/leaving, changes in the policy landscape, and resources coming on/offline. All of these factors were also influential in the development of MT in schools. Being able to recognize these and capitalize on them appears to be the key to effective implementation and where all four cornerstones come into play together.

### Limitations and Future Research

We adopted a case study design, drawing qualitative data from seven UK schools. While this approach has strengths in providing a rich understanding of the perceptions of stakeholders within different schools concerning the process of implementation of MT within their settings, it also has limitations. For example, although initial coding was checked by two additional team members, and initial findings were subject to member checking, there is still potential for interpretation of the data to be affected by the unconscious biases of the researchers involved. Further, because our selection of focus group members within schools was conducted through the snowball technique, and involved the mindfulness lead, relationships with and/or perceptions of the mindfulness lead at the school could have affected other teachers’ willingness to participate, and/or the nature of their participation. We did not quantify our findings and cannot therefore provide accurate estimations of the number of staff members within the participant pool who referenced particular themes or subthemes. However, because our focus groups were deliberately sampled to include a range of perspectives, such quantification would not necessarily provide an accurate picture of the distribution of opinions within the wider school communities from which participants were drawn. Rather our intention was to identify key themes relevant to understanding the journey towards implementation of MT in a range of UK schools.

An additional limitation concerns the fact that the proportion of schools currently engaging with mindfulness training in any systematic way in the UK is still low, and identification of such schools was challenging, restricting the sample of schools that could be included in the study. Ultimately, we included around half the schools that we were able to identify that had ongoing systematic provision of MT, and these schools reflected a broad range of school types. MT in UK schools is still in its early stages and follow-up research of our study schools would be valuable, to examine how initial findings concerning the school implementation context related to the later progression of the schools’ implementation journeys. This is particularly the case because we included schools at very different stages in their implementation journey. While this has advantages, such as enabling us to access the perspectives of staff in schools that may be struggling to implement MT and may not ultimately develop and sustain MT provision, it also has limitations. In particular, some participants and schools may not yet have encountered, and thus may not be able to report upon, the full range of circumstances that support and hinder implementation. Finally, we did not gather qualitative data from parents and carers or young people within the schools and nor did we collect quantitative data on aspects of the implementation process. These additional perspectives would have added an enriching dimension to the work, and some will be addressed through our ongoing research.

Despite these limitations our study also has strengths. There is very little work exploring the implementation of MT in schools, despite the fact that it is recognized as an important focus for future development of the field. Our findings suggest that implementation of MT in schools is an ongoing, non-linear process, with a range of factors influencing the implementation journey in each school. However, despite these unique journeys, in every case, institutional resilience and patience are likely to be required to allow the initial introduction of MT to develop and grow into sustainable provision.

## Supplementary Material

**Electronic supplementary material** The online version of this article (https://doi.org/10.1007/s12671-018-0982-4) contains supplementary material, which is available to authorized users.

Supplementary material

Appendices

## Figures and Tables

**Table 1:** School sociodemographic characteristics, number of staff in study, and duration of MT implementation within each school

School pseudonym	School type	Location within England	School size	Quality rating	Pupil attainment at 16	Pupil deprivation	Pupils w/ English as additional language	Number of staff in study	Duration of MT implementation within school
Meadow	State-Funded Academy	Rural Southwest	Large	Good	Average	Below Average	Below Average	14	3.5 years
Park	State-Funded Academy	Urban Southeast	Small	Requires improvement	Not Available	Above Average	Above average	9	2 years
Lake	Independent	Urban Midlands	Small	n/a	Above Average	Not reported	Not Reported	9	~ 1 year
Fields	Independent	Urban South	Large	n/a	Above average	Not Reported	Not Reported	8	8 years
Leafy	Independent	Suburban Southeast	Small	n/a	Above average	Not Reported	Not Reported	27	8 years
Garden	State-Funded Selective	Suburban Northwest	Large	Outstanding	Above average	Below average	Below average	5	Several years, patchy
River	State-Funded Academy	Urban Midlands	Small	Good	Below average	Above average	Above average	6	Several years, patchy

The schools took part on the basis that they would be anonymized and so are given pseudonyms throughout and school data are approximated to protect anonymity. School size is small if under 1000 pupils and large if over 1000 pupils. School inspection ratings for state schools were obtained from the Office for Standards in Education (OFSTED), independent schools are not rated by OFSTED so quality data is not available for these schools. Free school meal eligibility is assessed according to proportion percentage eligible for free school meals within previous 6 years and compared against the national average. Attainment is assessed against national performance indicators for qualifications taken at age 16 and comparedwith the same-year national average. Percentage of pupils with English as additional language is compared to the same-year national average

**Table 2:** Characteristics and mindfulness experience of participants within each school

School pseudonym	Participating staff (gender)	School role of interviewees (I) and focus group members	Participant experience of mindfulness and teaching MT in schools
Meadow	14 (seven male, seven female)	Head teacher (I), two deputy head teachers (I), the mindfulness lead (I), and a Special Educational Needs Coordinator (SENCO) (I) Focus groups included staff who taught across a range of subjects in arts, sciences and humanities	Four members of staff had no personal experience of mindfulness and at least two were actively skeptical. One member of staff had done a personal 8-week mindfulness course but had not continued to engage actively with mindfulness within the school. The remaining nine participants had done a personal 8-week mindfulness course and then continued to use mindfulness in their personal and professional lives, including informing their interactions with students. However, at the time of the research only the mindfulness lead was formally delivering MT within the school
Park	Nine (two male, seven female)	Head teacher (I), two deputy head teachers (I) and mindfulness lead (I) Focus groups comprised members of staff who held a range of teaching and pastoral support roles within the school	All of the staff had attended and introduction to mindfulness session and five had done a personal 8-week mindfulness course. The mindfulness lead was teaching mindfulness to pupils, parents and staff in the school. One other member of staff was trained to deliver MT to pupils but was not doing so at the time of this research
Lake	Nine (all female)	Head teacher (I), two mindfulness leads (I) The focus group included staff teaching a range of subjects including humanities, sciences and physical education as well as members of pastoral staff	The two mindfulness leads were trained to teach MT to pupils. The remaining staff had little exposure to MT and had not done personal mindfulness training
Fields	Eight (all male)	Head teacher (I), mindfulness lead (I) The focus group included staff teaching across the humanities, sciences and physical education	All members of staff had some exposure to mindfulness through the school culture, all had observed mindfulness practices being taught within the school and a large proportion of overall teaching staff had either done personal 8-week mindfulness course or a taster session. The mindfulness lead and one other member of staff were trained and currently teaching MT to pupils at the school
Leafy	27 (20 males, 7 females)	Head teacher (I), three members of the Senior Leadership Team (I) Focus groups included three members of pastoral staff, two members of support staff and a range of teaching staff across the sciences, arts and humanities	10 staff members had completed a personal 8-week mindfulness course, one had observed a pupil course, one had a personal meditation practice and another attended voluntary drop-in sessions within the school. Two had taught MT to pupils within the school. The majority had some exposure to mindfulness practices as part of the school culture
Garden	Five (two males, three females)	Head teacher (I), mindfulness lead (I) The focus group included three members of staff who taught across a range of curriculum areas	The mindfulness lead, and one other teacher had trained to deliver the MT curriculum. Levels of exposure to mindfulness amongst the other three teachers were unclear
River	Six (three males, three females)	Head teacher (I) Focus groups included teachers across a range of curriculum areas	Two participants had undertaken some personal mindfulness training and were teaching mindfulness skills as part of the school curriculum, having adapted some published resources. The other four participants had not completed a mindfulness course and did not teach mindfulness in the school

The schools took part on the basis that they would be anonymized and so are given pseudonyms throughout. Information about participant roles within schools and mindfulness experience is summarized to provide an overview of the characteristics of participants within each focus group, without including information that might be identifying

## References

[R1] BERA (2011). Revised Ethical Guidelines for Educational Research. http://www.bera.ac.uk/files/2011/08/BERA-Ethical-Guidelines-2011.pdf.

[R2] Braun V, Clarke V (2006). Using thematic analysis in psychology. Qualitative Research in Psychology.

[R3] Cooper BR, Bumbarger BK, Moore JE (2015). Sustaining evidence-based prevention programs: Correlates in a large-scale dissemination initiative. Prevention Science.

[R4] Dariotis JK, Mirabal-Beltran R, Cluxton-Keller F, Gould LF, Greenberg MT, Mendelson T (2017). A qualitative exploration of implementation factors in a school-based mindfulness and yoga program: Lessons learned from students and teachers. Psychology in the Schools.

[R5] Dimidjian S, Segal ZV (2015). Prospects for a clinical science of mindfulness-based intervention. American Psychologist.

[R6] Domitrovich CE, Bradshaw CP, Poduska JM, Hoagwood K, Buckley JA, Olin S (2008). Maximising the implementation quality of evidence-based preventative interventions in schools: A conceptual framework. Advances in School Mental Health Promotion.

[R7] Durlak JA (2002). Evaluating evidence-based interventions in school psychology. School Psychology Quarterly.

[R8] Durlak JA, Durlak J, Domitrovich CE, Weissberg RP, Gullotta TP (2015). What everyone should know about implementation. Handbook of social and emotional learning, research and practice.

[R9] Durlak JA (2016). Program implementation in social and emotional learning: Basic issues and research findings. Cambridge Journal of Education.

[R10] Durlak JA, DuPre EP (2008). Implementation matters: A review of research on the influence of implementation on program outcomes and the factors affecting implementation. American Journal of Community Psychology.

[R11] Durlak JA, Weissberg RP, Dymnicki AB, Taylor RD, Schellinger KB (2011). The impact of enhancing Students' social and emotional learning: A meta-analysis of school-based universal interventions. Child Development.

[R12] Eccles MP, Armstrong D, Baker R, Cleary K, Davies H, Davies S (2009). An implementation research agenda. Implementation Science.

[R13] Feagans Gould L, Dariotis K, Greenberg MT, Mendelson T (2016). Assessing fidelity of implementation (FOI) for school-based mindfulness and yoga interventions: A systematic review. Mindfulness.

[R14] Fixsen DL, Naoom SF, Blasé KA, Friedman RM, Wallace F (2005). Implementation research: A synthesis of the literature.

[R15] Greenberg MT (2010). School-based prevention: Current status and future challenges. Effective Education.

[R16] Greenhalgh T, Robert G, Bate P, Kyriakidou O, MacFarlane F (2005). Diffusion of innovations in health service organizations: a systematic literature review.

[R17] Grimshaw JM, Eccles MP, Lavis JN, Hill SJ, Squires JE (2012). Knowledge translation of research findings. Implementation Science.

[R18] Kabat Zinn J (2003). Mindfulness-based intervention in context: Past, present, and future. Clinical Psychology: Science and Practice.

[R19] Kitson AL, Rycroft-Malone J, Harvey G, McCormack B, Seers K, Titchen A (2008). Evaluating the successful implementation of evidence into practice using the PARiHS framework: Theoretical and practical challenges. Implement Science.

[R20] Klingbeil DA, Renshaw TL, Willenbrink JB, Copek RA, Chan KT, Haddock A (2017). Mindfulness-based interventions with youth: A comprehensive meta-analysis of group design studies. Journal of School Psychology.

[R21] Lincoln YS, Guba EG (1985). Naturalistic enquiry.

[R22] Maynard BR, Solis M, Miller V, Brendel KE (2017). Mindfulness-based interventions for improving cognition, academic achievement, behavior and socio-emotional functioning of primary and secondary students. Campbell Systematic Reviews.

[R23] Meiklejohn J, Phillips C, Freedman ML, Griffin ML, Biegel G, Roach A (2012). Integrating mindfulness training into K-12 education: Fostering the resilience of teachers and students. Mindfulness.

[R24] Merry SM (2012). Preventing depression in adolescents. British Medical Journal.

[R25] Miles MB, Huberman AM, Saldaña J (2014). Qualitative data analysis: A methods sourcebook.

[R26] Nilsen P (2015). Making sense of implementation theories, models and frameworks. Implementation Science.

[R27] Pearson M, Chilton R, Wyatt K, Abraham C, Ford T, Woods HB, Anderson R (2015). Implementing health promotion programs in schools: A realist systematic review of research and experience in the United Kingdom. Implementation Science.

[R28] Rycroft-Malone J, Bucknall T (2010). Models and frameworks for implementing evidence-based practice: Linking evidence to action.

[R29] Rycroft-Malone J, Anderson R, Crane R, Gibson A, Gradinger F, Owen-Griffiths H (2014). Accessibility and implementation in UK services of an effective depression relapse prevention program—mindfulness-based cognitive therapy (MBCT): ASPIRE study protocol. Implementation Science.

[R30] Rycroft-Malone J, Gradinger F, Griffiths HO, Crane RS, Gibson A, Mercer S (2017). Accessibility and implementation in UK services of an effective depression relapse prevention program: Learning from mindfulness-based cognitive therapy through a mixed-methods study. Health Services and Delivery Research.

[R31] Saldaña J (2012). The coding manual for qualitative researchers.

[R32] Sandelowski M, Barroso J (2007). Handbook for synthesizing qualitative research.

[R33] Sawyer SM, Afifi RA, Bearinger LH, Blakemore SJ, Dick B, Ezeh AC, Patton GC (2012). Adolescence: A foundation for future health. Lancet.

[R34] Stake R (1995). The art of case study research.

[R35] Tracy SJ (2010). Qualitative quality: Eight “Bbig-tent” criteria for excellent qualitative research. Qualitative Inquiry.

[R36] Vostanis P, Humphrey N, Fitzgerald N, Deighton J, Wolpert M (2013). How do schools promote emotional well-being among their pupils? Findings from a national scoping survey of mental health provision in English schools. Child and Adolescent Mental Health.

[R37] Weare K, Nind M (2011). Mental health promotion and problem prevention in schools: What does the evidence say?. Health Promotion International.

[R38] Yin RK (2014). Case study research design and methods.

[R39] Zenner C, Herrnleben-Kurz S, Walach H (2014). Mindfulness-based interventions in schools-a systematic review and meta-analysis. Frontiers in Psychology.

