# Supplementary material for: Mindfulness Training in UK Secondary Schools: a Multiple Case Study Approach to Identification of Cornerstones of Implementation
Source: Mindfulness (N Y). Author manuscript; Available in PMC 2019 Jun 11. (PMC6558285; doi:10.1007/s12671-018-0982-4)
Supplement: Supplementary material [file EMS83164-supplement-Supplementary_material.docx]

**Proposed Online Supplementary Material**

**Full case descriptions for each study school**

**Meadow** was a large, co-educational, state-funded academy school with pupils aged 11-18 in a rural setting in the South West of England, with an inspection rating of ‘good’. On average, pupils were high-achieving as compared to the national average. The number of pupils eligible for free school meals was very close to the national average and a very small proportion had English as an additional language when compared to the national average. This school had been working on implementing MT for about 3.5 years. Twenty-five to 30 members of staff had completed personal mindfulness training in this time. Only the mindfulness lead had additionally completed syllabus training in order to deliver MT to pupils, although a number of other staff members were interested in completing this training. MT for pupils was delivered primarily through drop-in sessions and through shorter sessions delivered to whole classes during registration periods. Specific groups of pupils (e.g. those with SEN or anxiety) were also identified to receive MT, and the mindfulness lead also led groups for parents and staff after school. In this school we interviewed 14 individuals: three members of the SLT, including the Headteacher, the mindfulness lead, and nine members of staff in a focus group. One additional member of staff was interviewed individually as they had responsibility for delivering mindfulness to pupils with special educational needs as part of a separate stream of teaching and learning in the school.

**Park** was a large, co-educational, state-funded academy school with pupils aged 11-18 in an urban area in the South East of England, with an inspection rating of ‘requires improvement’. There was a high level of deprivation and a far higher percentage of pupils spoke English as an additional language compared with the national average. Data on academic attainment was unavailable as this was a relatively new school. This school had been working on implementing MT for approximately two years. The mindfulness lead and one other trained member of staff delivered MT. In the initial stages of implementation MT had been delivered in half-hour slots in year 8 (pupils age 12-13). However, more recently MT had become an after-school option, as well as being delivered in drop-in sessions during exam periods and as an intervention for particular students, reducing overall reach. The mindfulness lead also ran MT groups for staff and parents. In this school we interviewed nine individuals: two members of the SLT, including the Headteacher, the mindfulness lead, and six members of staff in a focus group.

**Lake** was a small, single-sex, independent school with pupils aged 11-18 in an urban area in the Midlands. Lake had a very high level of academic attainment. Further data on the school characteristics were either unavailable or not applicable due to this being an independent school. This school had two trained Mindfulness Leads but was new to implementing MT, with each lead having only taught one 10 week MT course to a year 10 class in a timetabled slot assigned to Personal Social, Health and Economic Education. A further four courses for year 10 (pupils age 14-15) classes were planned, alongside a lunchtime club, as a way of sustaining MT. In this school we interviewed nine individuals: three members of the SLT, including the Headteacher, the mindfulness lead, and five members of staff in a focus group.

**Fields** was a large independent single-sex school with pupils aged 11-18 in an urban area in the South of England. Fields had a very high level of academic attainment. Staff at this school had been working on implementing MT for eight years and in this time the school had two mindfulness leads. Five members of staff were currently trained to deliver the MT curriculum used within the school. Fifty-eight members of staff had been through an eight-week personal mindfulness training course and a further 41 had done a taster session. More staff members were intending to train to deliver the MT course to students. Since 2012/2013 a full 10 week MT course had been delivered to all students when in Year 10. In this school we interviewed eight individuals: the Headteacher, the mindfulness lead, and six staff members in a focus group.

**Leafy** was a small, single-sex, independent school with pupils aged 13-18 in the suburban South East region of the England. Further data on the school characteristics were either unavailable or not applicable due to this being an independent school. This school had been working on implementing MT for around eight years, and in the two years prior to the fieldwork at least 80% of pupils in Year 10 (pupils aged 14-15) had received MT during core curriculum time. Four teachers were trained to deliver MT, including the head teacher of the school. In this school we interviewed 27 individuals: four members of the SLT including the Headteacher, and 23 members of staff across a series of three focus groups. In this school the mindfulness lead supported identification of staff and described the school’s implementation journey informally, but did not participate in the interviews or focus groups.

**Garden** was a large, single-sex, selective, state-funded grammar school with pupils aged 11-18, located in an suburban setting in the North West, with an inspection rating of ‘outstanding’ and a very high level of academic attainment. The level of deprivation was very low and the number of pupils who spoke English as an additional language was below the national average. The school had had a very effective mindfulness lead, who had since left the school. A previous headteacher had also shown an interest in mindfulness and had encouraged staff to train to deliver MT to pupils. At the time of the interview two teachers were trained to deliver MT, which was offered to all pupils in year 10 (pupils aged 14-15). Three further teachers had been trained but had either left the school or were about to leave and a new teacher was intending to train to teach MT over the summer. It was estimated that in this school around 20-25% of staff had completed an 8-week personal mindfulness course. In this school we interviewed five individuals: the Headteacher, the mindfulness lead, and three members of staff in a focus group.

**River** was a small, co-educational, state-funded academy with pupils aged 11-18 with a ‘good’ inspection rating, located in an urban area in the Midlands. The academic attainment of pupils was above the national average, and the level of deprivation was high. Around half of the pupils spoke English as an additional language. Implementation at this school had been unsystematic. A former member of staff who had been trained to deliver MT had taught an established course within the school but had subsequently left. At one point seven teachers within the school had completed personal mindfulness training, but by the following year five of these staff members had left. The head teacher was supportive of MT but did not anticipate training more staff due to cost constraints. At this school we interviewed the Headteacher, and five members of staff participated in a focus group. There was no identified mindfulness lead. At this school we interviewed six individuals: the Headteacher, and five members of staff participated in a focus group.
