# Supplementary material for: Mindfulness Training in UK Secondary Schools: a Multiple Case Study Approach to Identification of Cornerstones of Implementation
Source: Mindfulness (N Y). Author manuscript; Available in PMC 2019 Jun 11. (PMC6558285; doi:10.1007/s12671-018-0982-4)
Supplement: Appendices [file EMS83164-supplement-Appendices.pdf]

- What evaluation of mindfulness teaching occurs in the school?
- Is there succession planning for mindfulness in the school?
- Is there provision of mindfulness training for other members of school staff?
- Is there provision of mindfulness training for parents?

What have been the main barriers and facilitators to introducing mindfulness into your school?

- What has helped to introduce mindfulness in your school?
- Which challenges has the school faced in introducing mindfulness?

What could support or hinder schools in the future?

- What kinds of support would help your school deliver mindfulness in the future?
- What could work against the sustainable use of mindfulness at your school?

Appendix 1

Interview schedule for senior leadership and mindfulness lead teachers

What is the implementation narrative at the school? What are the perceived purposes of MT implementation in school settings?

- How did your school become involved in mindfulness? Which people have played a key role in bringing mindfulness to your school?
- What were some of the key milestones?
- What are the main reasons for bringing mindfulness into your school? How does mindfulness fit with your school's priorities?

Probes:

- How is mindfulness taught in the school?
- Which pupils/year groups receive mindfulness teaching in the school?
- How many members of staff teach mindfulness at the school?
- What training has the mindfulness lead (/teachers) completed to support their teaching of mindfulness?

Implementation guidance

- MYRIAD will produce implementation guidance for schools wishing to introduce mindfulness. What information or resources could have helped you in the earlier stages of the journey to bringing mindfulness into your school?

Appendix 2

Focus group interview schedule

What are the main challenges and facilitators for mindfulness in schools?

Invitation to discuss these in small groups and write them down on posters and sticky notes.

- What has helped your school to implement mindfulness?
- What challenges has your school faced in introducing mindfulness?
- What criticism has there been of mindfulness at the school?

What could support your school to work with mindfulness in the future? What could create challenges?

- What are the three most important facilitators for bringing mindfulness into your school?
- What are the three biggest challenges?
- How does mindfulness fit with your school's priorities?
- What kinds of support would you appreciate for mindfulness in your school in the future?

Implementation guidance

- MYRIAD will produce implementation guidance for schools wishing to introduce mindfulness. What information or resources could have helped you in the earlier stages of the journey to bring mindfulness into your school? Would you prefer online resources, printed resources or both?

Open Access This article is distributed under the terms of the Creative Commons Attribution 4.0 International License (http://creativecommons.org/licenses/by/4.0/), which permits unrestricted use, distribution, and reproduction in any medium, provided you give appropriate credit to the original author(s) and the source, provide a link to the Creative Commons license, and indicate if changes were made.
